# Facilitating long-term cell examinations and time-lapse recordings in cell biology research with CO_2_ mini-incubators

**DOI:** 10.1038/s41598-024-52866-y

**Published:** 2024-02-10

**Authors:** Ali Talebipour, Mehrdad Saviz, Mohaddeseh Vafaiee, Reza Faraji-Dana

**Affiliations:** 1 Department of Biomedical Engineering, Amirkabir University of Technology (Tehran Polytechnic), Tehran, Iran; 2 Institute for Nanoscience and Nanotechnology, Sharif University of Technology, Tehran, Iran; 3 Center of Excellence on Applied Electromagnetic Systems, School of Electrical and Computer Engineering, College of Engineering, University of Tehran, Tehran, Iran

**Keywords:** Cell biology, Developmental biology, Drug discovery, Molecular biology

## Abstract

In recent years, microscopy has revolutionized the study of dynamic living cells. However, performing long-term live cell imaging requires stable environmental conditions such as temperature, pH, and humidity. While standard incubators have traditionally provided these conditions, other solutions, like stagetop incubators are available. To further enhance the accessibility of stable cell culture environments for live cell imaging, we developed a portable CO_2_ cell culture mini-incubator that can be easily adapted to any x–y inverted microscope stage, enabling long-term live cell imaging. This mini-incubator provides and maintains stable environmental conditions and supports cell viability comparable to standard incubators. Moreover, it allows for parallel experiments in the same environment, saving both time and resources. To demonstrate its functionality, different cell lines (VERO and MDA-MB-231) were cultured and evaluated using various assays, including crystal violet staining, MTT, and flow cytometry tests to assess cell adhesion, viability, and apoptosis, respectively. Time-lapse imaging was performed over an 85-h period with MDA-MB-231 cells cultured in the mini-incubator. The results indicate that this device is a viable solution for long-term imaging and can be applied in developmental biology, cell biology, and cancer biology research where long-term time-lapse recording is required.

## Introduction

In vitro cell culture has revolutionized the field of biomedical research by enabling the growth and manipulation of cells outside of their natural environment. Despite the absence of a supporting body infrastructure, in vitro cell culture has proven to be a valuable tool for researchers to model and investigate functional physiological subsets^1–3^.

In fact, cell culture has become indispensable in various areas of research, including drug discovery^4^, regenerative medicine^5^, cancer research^6^, and virology. One significant advantage of cell culture is its utility in drug discovery. Cell-based assays have become the standard for evaluating the efficacy and toxicity of potential drug candidates. By growing cells in vitro, researchers can screen a large number of compounds quickly and efficiently, providing valuable information about the potency and specificity of potential drugs. In regenerative medicine, cell culture plays a pivotal role in propagating and manipulating different types of cells for therapeutic purposes. Researchers can create three-dimensional tissue constructs that mimic the anatomy and physiology of intact organs, which can then be used for transplantation or as a platform for drug testing. In cancer research, cell culture has been instrumental in elucidating the fundamental molecular and cellular mechanisms underlying cancer development, progression, and response to therapies. By growing cancer cells in vitro, researchers can study their behavior under different conditions, identify potential drug targets, and test new therapies. Finally, in the context of the COVID-19 pandemic, cell culture has played a crucial role in advancing our understanding of SARS-CoV-2^7^. Researchers have used cell culture to study the cellular processes involved in virus-host interactions, develop antiviral drugs, and test vaccine candidates. Overall, in vitro cell culture has enabled unprecedented advances in biomedical research and holds tremendous promise for future developments in fields ranging from regenerative medicine to drug discovery and beyond.

In recent years, the field of cell culture has seen a significant shift towards more advanced and sophisticated techniques that require precise control and monitoring of environmental conditions. One such technique is time-lapse imaging, which has emerged as a cutting-edge technique in the field of cell biology, offering researchers an unprecedented window into dynamic cellular behaviors^8^.

With this cutting-edge technique, researchers can continuously monitor cells over time and capture their behavior in real-time, providing valuable insights into cellular processes such as cell migration^9^, proliferation^10^, differentiation^11^, and more. This powerful tool has revolutionized the field of cell biology, providing a deeper understanding of cellular behavior and its underlying mechanisms. However, maintaining long-term vital conditions such as temperature, carbon dioxide (CO_2_) concentration, and humidity can be challenging and, in some cases, not easily adaptable to conventional inverted microscopes.

The cell incubator is a commonly used device in cell-biology labs, designed to provide an environment that is conducive to the growth, survival, and proliferation of cells. This typically involves maintaining a constant temperature of 37 °C, a 5% concentration of CO_2_ to stabilize the pH of cell culture media, and a relative humidity of about 90–95% to minimize water evaporation. However, cell incubators are often incompatible with many screening tools, such as microscopy and electrophysiology, which limits experimental possibilities. There has been a trend towards delivering essential flows (air, heat, humidity, etc.) to the microscope stage for long-term observations, while ambient conditions are sometimes utilized for short experiments (< 5 h). Ambient conditions pose challenges in terms of cell culture temperature, contamination, and mechanical disturbances. Additionally, the lower CO_2_ content in the environmental air compared to the ideal percentage in an incubator can rapidly elevate the pH of the cell culture medium. Furthermore, the evaporation of the medium due to the unsaturated moisture content of the environmental air may induce hyperosmolarity. These issues are exacerbated when experiments are conducted over longer durations, from hours to days. To address these issues, several strategies have been developed, including the integration of optical microscopes into systems that control all the required conditions. However, these systems tend to be large, and bulky, and enclose the entirety of the microscope, making it difficult to assemble and remove between uses, hindering the general use of the microscope, and often being expensive. Another strategy is the development of smaller incubators that can be positioned on top of the microscope stage, providing a more portable and adaptable solution to different microscopes. These mini-incubators are specifically designed to provide a stable and controlled environment for cell growth while being compact and portable. They offer an ideal solution for researchers who require precise environmental control but do not have access to a larger lab incubator or wish to perform experiments with the integration of other imaging, stimulation, and recording equipment^12–15^. With such innovative solutions, we can expect to see continued progress in the field of cell biology with new insights and discoveries that were previously impossible to achieve.

While commercial and custom-made microscope incubators have shown results in stabilizing the pH, temperature, and humidity of cell cultures, they are not without their limitations. One of the primary drawbacks of these incubators is their bulkiness^16^, which can make them difficult to handle and move around. In addition, some custom-made microscope incubators use heating elements adjacent to the cell chamber^17,18^, which can create electrical and magnetic interference that may impact experimental outcomes, especially in bioelectromagnetics research. These issues can be particularly problematic when conducting long-term cell examinations or time-lapse recordings, which require extended periods of stable environmental conditions. Therefore, there is a growing need for a more efficient solution that can overcome these limitations and provide a stable, controlled environment without adding any extra interference to the experiment.

In this study, we have developed a portable on-stage mini-incubator for cell culture that utilizes a microcontroller. The mini-incubator is specifically designed to be easily adaptable to any x–y stage of a conventional inverted microscope, making it suitable for long-term time-lapse imaging while maintaining optimal conditions such as temperature, CO_2_ concentration, and humidity through feedback control systems. One key advantage of this mini-incubator system is that it is free from any electrical and magnetic interference, providing a stable and reliable environment for cell culture experiments. To ensure the accuracy and functionality of our mini-incubator, we performed several assays, including crystal violet staining, MTT, and flow cytometry. Overall, our mini-incubator represents a significant advancement in the field of cell culture, enabling researchers to conduct complex experiments with ease and precision.

## Materials and methods

### Mini-incubator system

**Figure 1:**
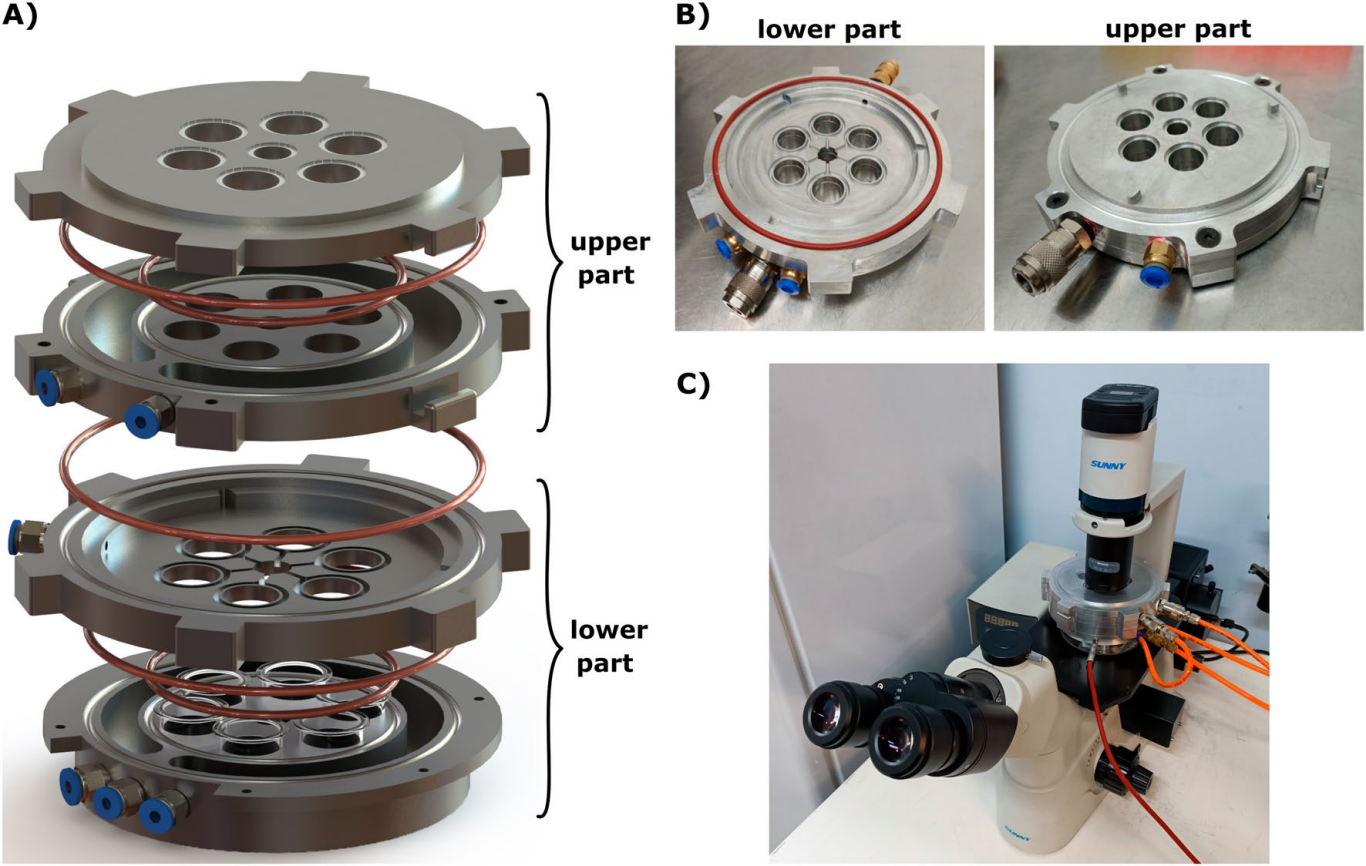
(**A**) Exploded view of the mini-incubator, showcasing the individual components that can be assembled using screws. (**B**) Assembled view of the two main parts of the mini-incubator. (**C**) Mini-incubator placed on an inverted microscope stage.

In this study, a 3D model of a mini-incubator was developed using SolidWorks software to achieve precise control over its dimensions and geometry. The resulting model was used to manufacture the mini-incubator through the use of CNC machining, utilizing 6061 aluminum alloy material for its construction. The mini-incubator is designed to be easily placed on a standard microscope stage. This feature allows the mini-incubator to be used for real-time or time-lapse imaging. Figure 1A shows the exploded view of the mini-incubator. The device uses silicone O-rings to prevent the entry of contaminants into the cell culture by creating a tight seal. The mini-incubator features six wells, with each well matching the size of a single well in a 24-well plate. This design allows for versatile usage, enabling researchers to conduct multiple parallel cultures or repeat experiments. The Assembled view of the mini-incubator is depicted in Fig. 1B,C. Additionally, CAD materials for the mini-incubator are provided in 'Sup3_CAD.pdf' in the supplementary materials.

**Figure 2:**
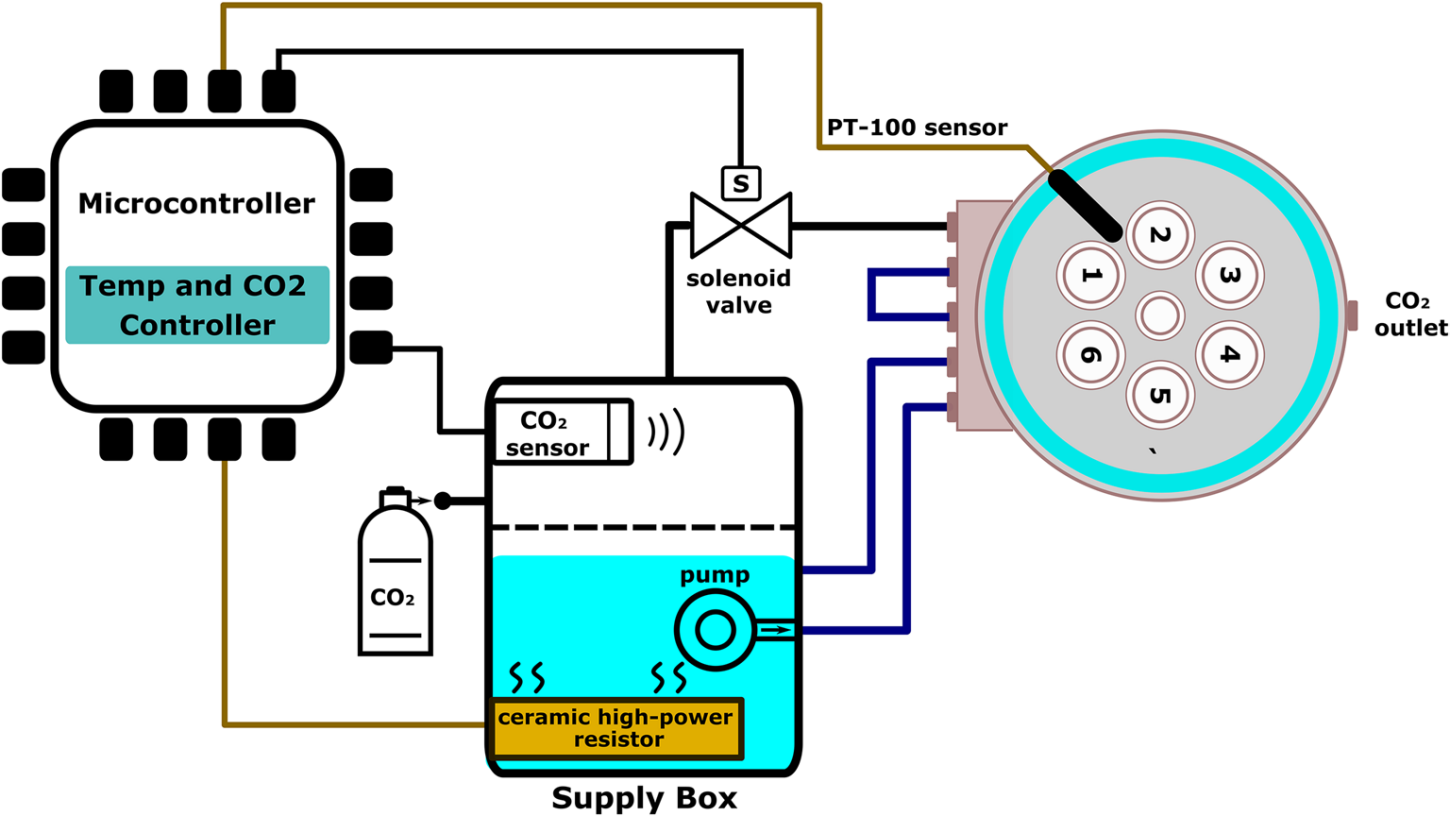
A simplified control diagram showcasing the utilization of a microcontroller for temperature, CO_2_ level, and humidity sensing and control.

Figure 2 shows the block diagram of the control system. To maintain the viability of most mammalian cells in culture, a temperature of 37 °C is essential, which is achieved by using water circulation around the mini-incubator (water jacket). The supply box provides water that is pumped into the water jacket by a water pump. Subsequently, the water flows around the device and eventually returns to the supply box via the outlet. To control the optimal temperature in the device, a microcontroller (ARM Cortex-M4 core) and 24-bit ADC (AD7124) are utilized in conjunction with ceramic high-power resistors (5 parallel resistors with a specification of 33 Ohms, 20W each) installed within the supply box, power supply (24V, 4.2A) and a PT-100 temperature sensor integrated into the mini-incubator body. The choice of aluminum as the construction material was based on its excellent thermal conductivity. This attribute, when combined with the water jacket, enables precise temperature control and uniformity within the mini-incubator.

In addition to temperature control, the mini-incubator also utilizes a sensor installed in the supply box and a solenoid valve to regulate the level of 5% CO_2_ gas, which is controlled by a microcontroller. The CO_2_ capsule contains a regulator that reduces pressure. As a result, the mini-incubator is supplied with humidified 5% CO_2_, facilitated by a water reservoir in the central chamber. This allows for a continuous flow of 5% CO_2_ throughout the device while maintaining appropriate humidity levels.

### Simulation

In order to optimize the fluid flow within the water jacket, simulations were carried out to determine the most effective path for the fluid. The simulations, performed using COMSOL software, analyzed the fluid velocity and temperature distribution within the mini-incubator. A fluid inlet velocity of 10 cm/s and a temperature of 39 °C were used in the simulation.

### Cell culture preparation

The MDA-MB-231 and VERO cell lines were purchased from the Iranian National Center for Genetic and Biologic Resources (Tehran, Iran). To prepare VERO and MDA-MB-231cells for experiments, DMEM/High glucose (Sigma) culture medium supplemented with 15% fetal bovine serum (Gibco) and 1% penicillin/streptomycin (Gibco) antibiotics was used. To passage the cells, the culture medium was aspirated, and the cells were washed with negative phosphate-buffered saline (PBS). Trypsin/EDTA enzyme was then added, and the cell culture plate was incubated at 37 °C for 1–4 min. After the incubation period, the enzyme was stopped by adding culture medium, and the cells were gently tapped until they detached from the bottom of the plate. The suspension was transferred to a Falcon tube and centrifuged at 770 rpm for 5 min or 1200 rpm for 3 min. The supernatant was aspirated, and the cells were resuspended in a freshly prepared culture medium and transferred to a flask or Petri dish. The culture medium was refreshed every 2–3 days to maintain the cells viability and ensure optimal growth. It is worth noting that the same protocol was followed for both VERO and MDA-MB-231 cells. Both cell types are essential in biomedical research for studying disease mechanisms, drug testing, and experimental assays in cancer biology and virology. MDA-MB-231 cells, established from a pleural effusion of a 51-year-old woman with metastatic breast cancer, are employed as models for breast cancer metastasis, while VERO cells, originating from the kidney of a normal adult African green monkey, find utility in virology and vaccine production.

### Cell viability

Two assays, the MTT assay and the Crystal Violet staining assay, were performed to assess cell viability. The MTT is based on the reduction of MTT by viable cells to form insoluble formazan crystals, which can be quantified by measuring their absorbance. On the other hand, the Crystal Violet staining assay is a straightforward and reliable method for assessing cell density and viability, where the dye binds to basic amino acid residues in cellular proteins, enabling the visualization and quantification of adherent cells.

For the MTT assay, the cell culture medium was removed from each well, and 50 µL of serum-free medium and 50 µL of MTT solution were added to each well. The MTT solution was prepared using MTT powder at a concentration of 10 mg/mL in water, 20 mg/mL in ethanol, and 5 mg/mL in fetal bovine serum. The wells were then incubated in the dark for 3 h at 37 °C. After incubation, 150 µL of DMSO was added to each well, and left for 10–15 min to dissolve. The absorbance at 570 nm and 630 nm wavelengths was measured using an ELISA reader.

To prepare the cells for staining with Crystal Violet, plates were placed on ice. Then, cells were washed twice with cold PBS and fixed with methanol at − 20 °C for 10 min. After removing the methanol and plates from the ice, enough Crystal Violet solution was added to the wells to dissolve the dye. The plates were then incubated for 10 min before removing the Crystal Violet solution and carefully washing the wells with distilled water to remove any excess dye. Finally, the plates were left at room temperature to dry.

### Flow cytometry

Flow cytometry was utilized in this study to analyze the cell culture. Firstly, the cells were washed with PBS to remove any residual culture media and external debris. This ensures a clean starting point for subsequent analyses. After washing, trypsinization was performed to detach the cells from the culture dish, allowing for the creation of a single-cell suspension. The cells were then counted to determine their concentration and ensure the appropriate number of cells for subsequent experiments. Following cell counting, FBS was added to the cell suspension to provide a protective environment and prevent cell aggregation or clumping during the analysis. The addition of FBS helps maintain the viability and integrity of the cells throughout the flow cytometry process. Finally, the prepared cell suspension was ready for further staining or analysis to assess cell apoptosis using flow cytometry techniques.

### Time-lapse recording

In order to observe the growth of VERO and MDA-MB-231 cells, a time-lapse imaging approach was employed in mini-incubator. Images were captured every 5 min for 4 days, with a single well selected as the reference for imaging. This allowed to track changes in cell morphology, proliferation, and behavior over time.

## Results

**Figure 3:**
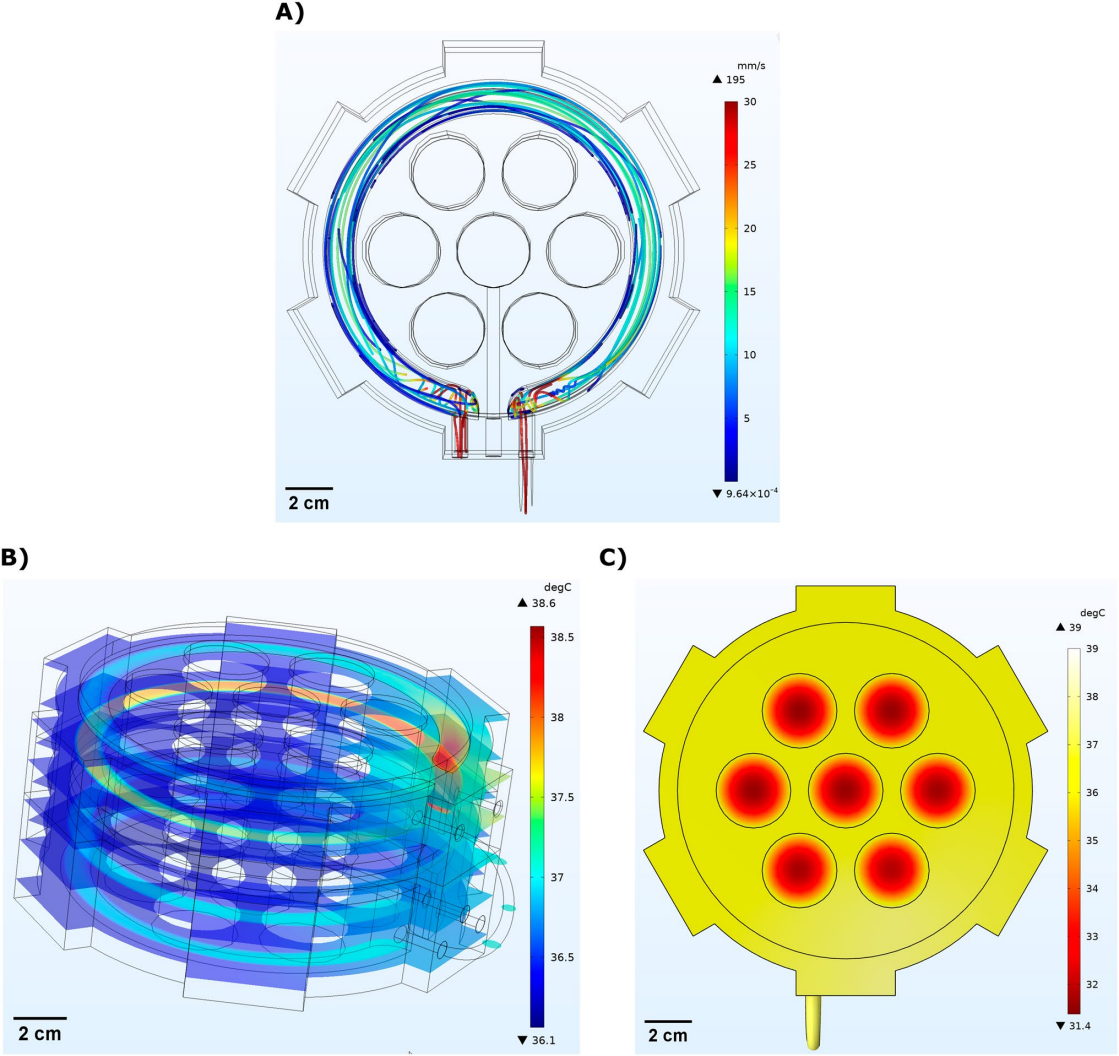
Overview of the simulation conducted for the mini-incubator device: (**A**) analyzing the fluid velocity in the water jacket, (**B**) examining the temperature distribution in the internal slices, and (**C**) evaluating the temperature distribution on the top surface of the mini-incubator.

The fluid velocity distribution obtained from the simulation is shown in Fig. 3A. The figure highlights the reduced presence of areas with very low speed and stagnant flow, which can be attributed to the circular design of the mini-incubator. Figure 3B,C depict the temperature distribution in the sliced sections and on the top surface (only top body and plexiglass) of the mini-incubator, respectively. The results demonstrate a relatively uniform temperature distribution across the top surface and internal components of the mini-incubator. However, certain parts exhibit slightly different temperatures, namely the plexiglass located in the upper portion of the mini-incubator and the fluid inlet region. The inner parts, particularly the surfaces containing the cell culture wells, maintain an approximately average temperature of 37 °C, consistent with our experimental findings. In order to validate the simulation results, we configured the temperature control to a setpoint of 37 °C. Subsequently, the water temperature inside the supply box reached 39 °C, as measured by a thermometer with an error margin of 0.1 °C. These findings confirm the reliability and precision of the mini-incubator's temperature control system, which consistently maintains a 2 °C differential compared to the water within the supply box. Results demonstrate that this design of mini-incubator is capable of providing a uniform and stable temperature distribution, making it a reliable tool for cell culture experiments.

The viability of VERO cells in the mini-incubator was assessed using the MTT assay and compared it to that of conventional incubator (INC 108, Memmert, Germany). Three wells from the incubator group and two wells from the mini-incubator group were analyzed using an ELISA device. Optical density readings were taken at 570 and 630 nm wavelengths, and the average of the three incubator wells was determined. Cell viability for each mini-incubator well was then calculated using the following Eq. ^19^:

$$Cell viability \left(\%\right)= \frac{{OD}_{570}\left(Mini-incubator\right)- {OD}_{630}\left(Mini-incubator\right)}{{OD}_{570}\left(incubator\right)- {OD}_{630}\left(incubator\right)}\times 100\%$$

**Figure 4:**
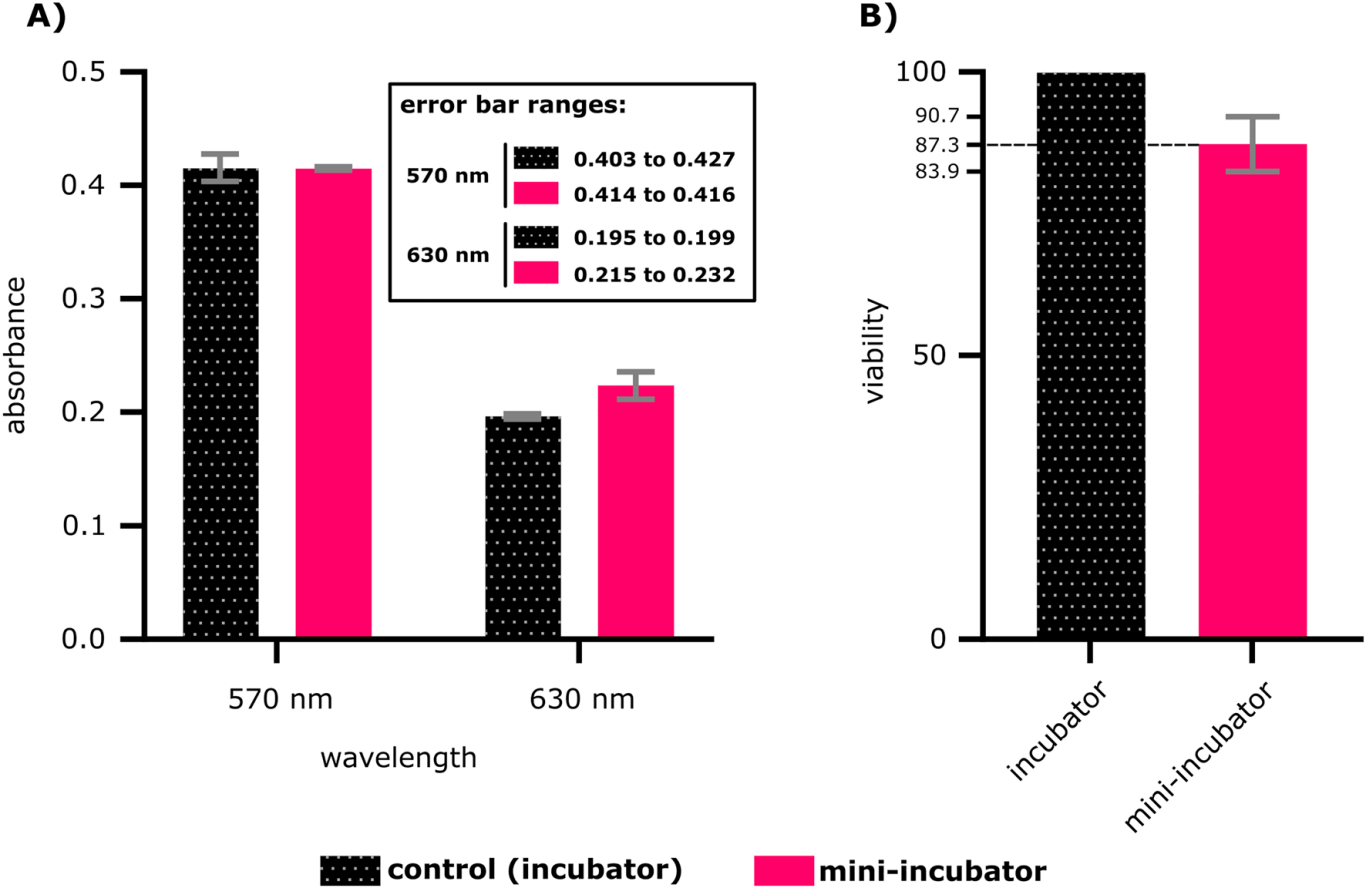
MTT assay results: (**A**) Optical density at 570 and 630 nm in incubator and mini-incubator. (**B**) Percentage of VERO cell survival in the mini-incubator compared to the conventional incubator. (*p*-value > 0.05, non-significant difference).

The results of the MTT assay are presented in the Fig. 4, showcasing the survival percentages of cells in both the mini-incubator and the incubator. The mini-incubator exhibits slightly lower cell survival percentages compared to the incubator. This difference is relatively small, suggesting that the mini-incubator is capable of supporting cell survival and growth at a comparable level to the incubator. These findings suggest that the mini-incubator can provide a suitable environment for cell culture, although further studies may be needed to fully assess its potential. The data obtained in this study also highlights the importance of evaluating the performance of new cell culture systems before their widespread use and provides valuable information for researchers who are considering using the mini-incubator for their experiments. The MTT assay results demonstrate that the mini-incubator is a promising alternative to conventional incubators for cell culture experiments.

**Figure 5:**
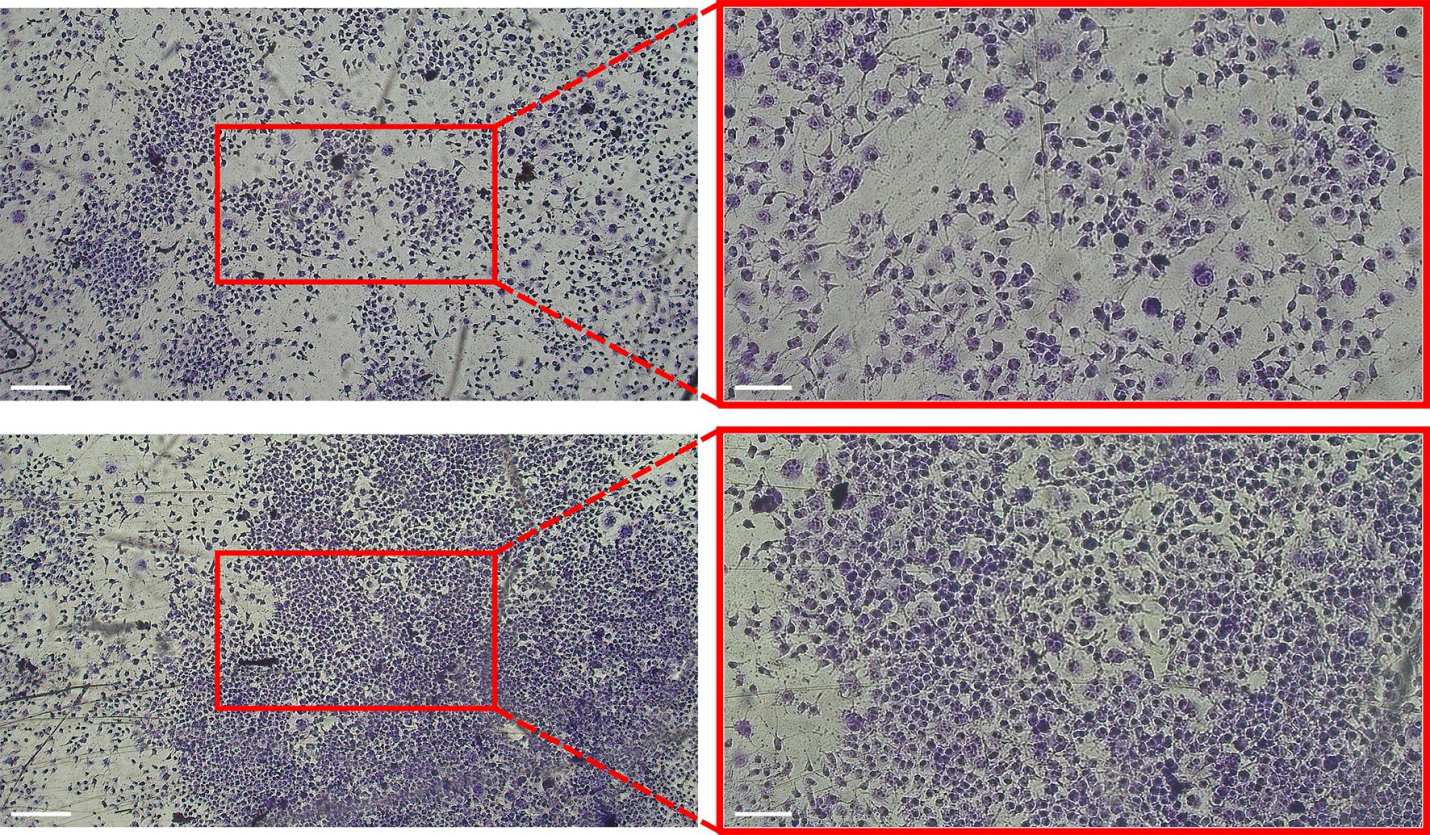
Crystal violet-stained MDA-MB-231 cells in two separate wells of the mini-incubator after 24 h of culture. The right images are shown at 4X magnification, with a scale bar of 500 µm, while the left images are at 10X magnification, with a scale bar of 200 µm.

MDA-MB-231 cells were cultured in the mini-incubator to evaluate its suitability for supporting cell growth and adhesion. After 24 h of culture, the cells were stained with crystal violet. Figure 5 shows images from cells cultured in two distinct wells within the mini-incubator. The qualitative analysis of the crystal violet-stained MDA-MB-231 cells in the mini-incubator revealed that the cells appeared healthy and well-organized in both wells, indicating that the mini-incubator was able to maintain the necessary conditions for cell growth and survival. The successful attachment and spreading of the cells further confirmed the suitability of the mini-incubator for cell culture. These findings are significant as they suggest that the mini-incubator could potentially be used as a cost-effective and efficient alternative to traditional cell culture methods, which can sometimes involve larger equipment and dedicated facilities.

**Figure 6:**
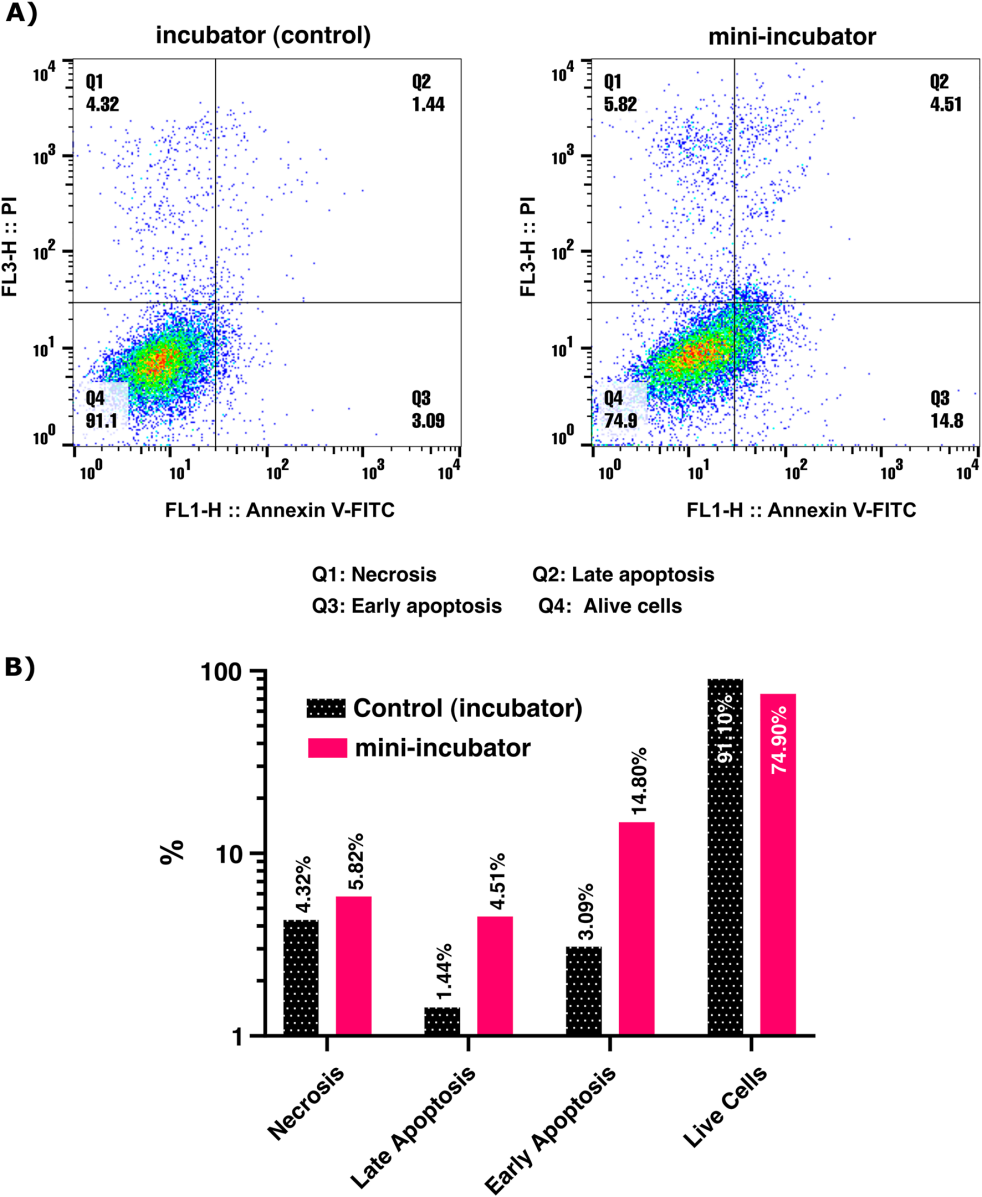
Results of flow cytometry analysis comparing apoptosis levels in (**A**) the mini-incubator and incubator and (**B**) their percentage. (*p*-value > 0.05, non-significant difference).

To investigate the effect of the mini-incubator on apoptosis levels in VERO cells, a flow cytometry experiment was conducted. Apoptosis, or programmed cell death, plays a crucial role in many biological processes, including tissue homeostasis and development. In this study, VERO cells were loaded for 4 days and subjected to flow cytometry analysis to compare apoptosis levels between the mini-incubator and control (incubator). To ensure an adequate sample size, four wells were pooled from each group separately. The analysis in FlowJo software was conducted on a total of 10,277 cells in the control group and 11,878 cells in the mini-incubator group (Fig. 6). The flow cytometry test differentiated between living cells, early apoptosis, late apoptosis, and necrotic cells^20^. The number of apoptotic cells (identified as Q2 + Q3, representing late and early apoptosis, respectively) in the mini-incubator group increased by 14.78% compared to the control group, as shown in the bar graph. The results of the flow cytometry experiment on VERO cells indicate that the mini-incubator had an effect on apoptosis levels in VERO cells. It's worth noting that further optimization of the mini-incubator's conditions and parameters could potentially lead to improvements in this aspect.

**Figure 7:**
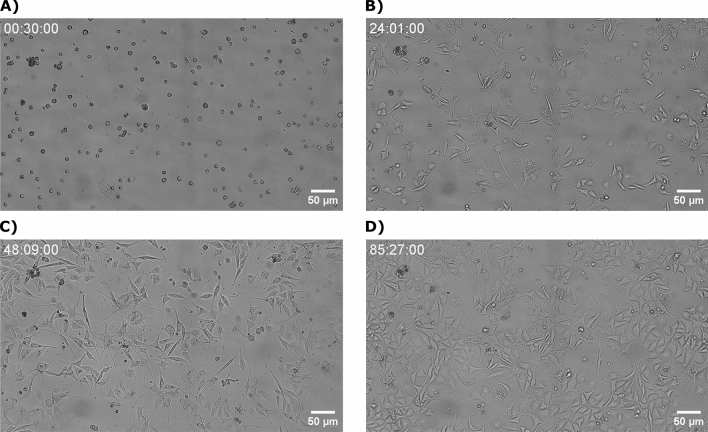
Time-lapse imaging of MDA-MB-231 cells at (**A**) 30 min, (**B**) 24 h, (**C**) 48 h, and (**D**) 85 h, demonstrating the progression of cell growth and division over time.

Time-lapse imaging is a powerful tool for verifying the performance of cell culture, as it enables continuous monitoring of cells over time, providing valuable insights into their behavior. In our study, we employed time-lapse imaging to investigate the performance of MDA-MB-231 and VERO cells cultured in a mini-incubator, with images captured at a 5-min time interval. The movies provided in the supplementary materials (Sup1-MDA_MB_231.mp4 and Sup2_VERO.mp4) allow for visual observation of cell migration and division, confirming the dynamic nature of the cells and their ability to proliferate in the mini-incubator. Time-lapse imaging also enabled us to track changes in cell morphology and behavior over time, providing a more comprehensive understanding of how the cells respond to their environment. Furthermore, the series of images presented in Fig. 7, captured at different time points, provide a clear demonstration of the cells' growth and division over an 85-h period, resulting in a cell density of approximately 80%. This level of cell growth and proliferation is comparable to what is typically observed in traditional incubators, further validating the suitability of the mini-incubator for conducting cell-based experiments. The ability to use time-lapse imaging to continuously monitor cells over time is a significant advantage, as it allows for real-time observation of cell behavior and changes, providing a more detailed understanding of the underlying mechanisms of cell growth and division. Moreover, time-lapse imaging can also help to identify any potential issues or problems that may arise during cell culture, such as contamination or cell death, allowing for timely intervention and troubleshooting.

These findings highlight the reliability and functionality of the mini-incubator for conducting cell-based experiments. Although further research is needed to fully characterize the mini-incubator's performance for different types of cells and applications, the results of our study provide a strong foundation for future investigations in this area.

**Figure 8:**
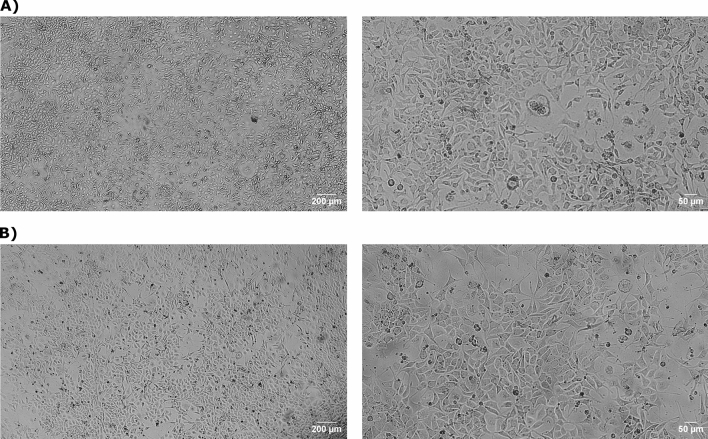
Qualitative comparison of MDA-MB-231 cells cultured in (**A**) incubator and (**B**) mini-incubator after 4 days, displayed at different magnification levels.

After 85 h of time-lapse imaging, images were captured from multiple wells in both the mini-incubator and the incubator to compare cell growth and proliferation. Figure 8 shows a side-by-side comparison of MDA-MB-231 cells cultured in both incubators at 4X and 10X magnification.

The results of this experiment indicate that the mini-incubator offers a promising environment for supporting cell growth and proliferation. These findings provide valuable insights into the use of mini-incubators as a potential alternative for cell culture, highlighting their potential advantages for different types of cells and experimental contexts. Further research in this area could lead to the development of more cost-effective and efficient methods for cell culture, with important implications for a wide range of biomedical research and applications.

## Discussion

The mini-incubator system, developed in this study, offers several advantages in comparison to other incubator systems. First, it is designed to be easily adaptable to any x–y stage of a conventional inverted microscope, making it suitable for long-term time-lapse imaging while maintaining optimal conditions such as temperature, CO_2_ concentration, and humidity through feedback control systems. This makes it a more portable and adaptable solution to different microscopes than larger custom-made microscope incubators. With the mini-incubator, extended observation periods are achievable, as demonstrated by our successful 85-h test involving two different cell types. It's worth noting that in both MTT assays and flow cytometry, we conducted Student's t-tests, which revealed that there is no statistically significant difference between the mini-incubator and the incubator. However, it is important to consider that cell viability in the mini-incubator should ideally be better for it to serve as a safe solution in cell culture. The reason for potential discrepancies is twofold. Firstly, the research involves a Laboratory Location Discrepancy, which may introduce variations. Secondly, we lack control over the internal Environmental Pressure, which can impact the results. In light of these challenges, we recommend researchers interested in building their mini-incubator to be mindful of these aspects. We are committed to optimizing and controlling these variables in our future work. Additionally, implementing a completely closed system for environmental control can aid in achieving the desired temperature in cell culture.

Another advantage of our mini-incubator system is that it is free from any electrical and magnetic interference, providing a stable and reliable environment for cell culture experiments. By incorporating a water jacket, it establishes an environment that eliminates interference and promotes uniform temperature distribution throughout the device. This is in contrast to some custom-made microscope incubators that use heating elements inside the system, which can create electrical and magnetic interference that may impact experimental outcomes, especially in bioelectromagnetics research. Additionally, our mini-incubator system is compact and easy to handle, making it ideal for use in small labs or in field research settings. It is also cost-effective compared to larger custom-made microscope incubators, which can be expensive and require specialized maintenance. In the context of maintenance, both the mini-incubator and larger incubators share common needs such as sensor calibration and sterilization. However, the distinguishing factor is the impact of size on these maintenance challenges. Larger incubators, due to their size, present greater difficulties in maintaining sterilization conditions and are associated with an increased risk of contamination. This is especially critical in environments where diverse cell lines are handled, leading to a higher potential for cross-contamination. The consequences of such contamination include substantial damage and elevated operational costs, issues that the mini-incubator, with its smaller scale, is better positioned to mitigate.

**Figure 9:**
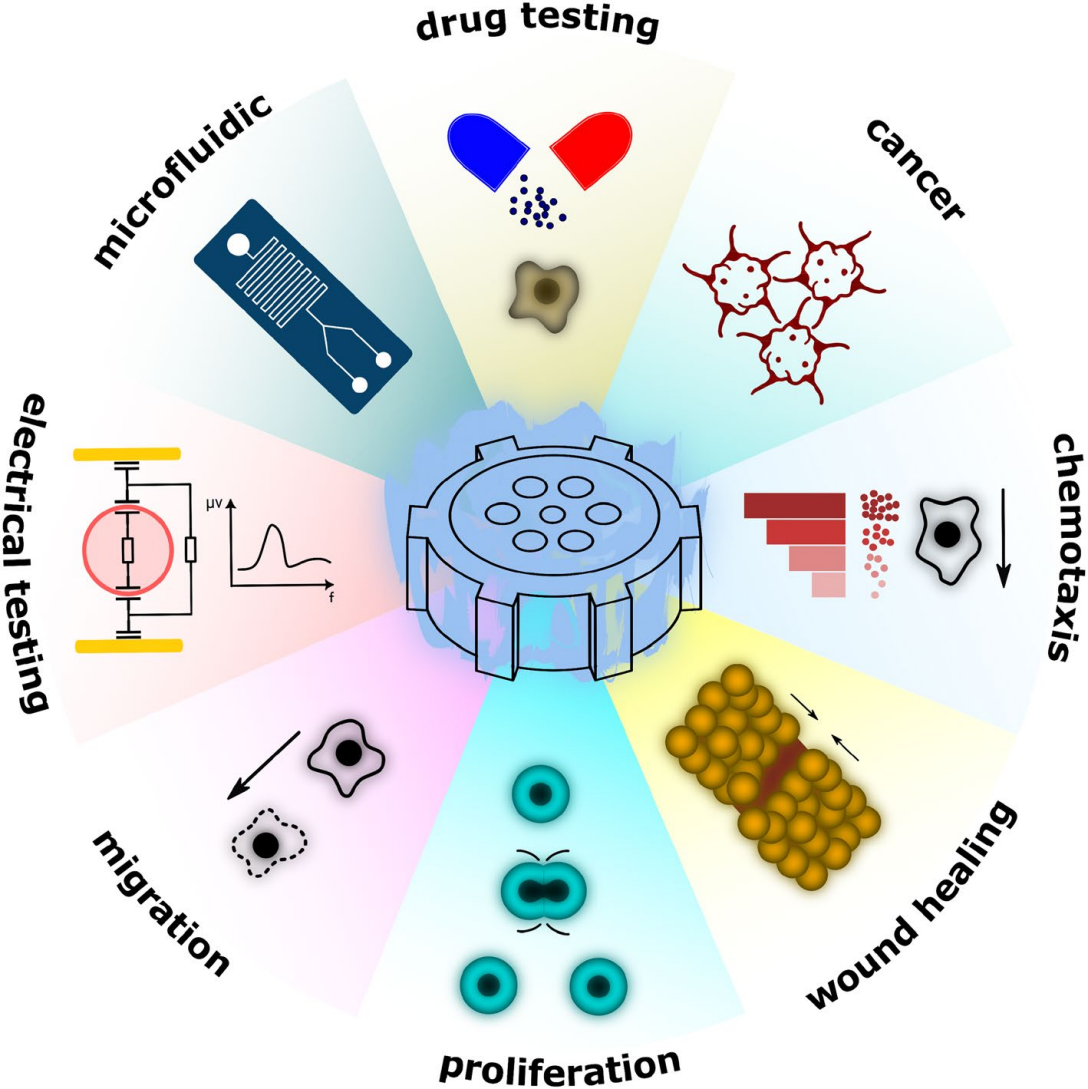
Applications of mini-incubators.

The mini-incubator has wide-ranging applications across various fields of research, including cancer biology, chemotaxis studies, drug testing, cell migration mechanisms, microfluidic experiments^21,22^, cell proliferation dynamics, electrical cell testing^23–25^, wound healing^26^ and etc. (Fig. 9). For example, the mini-incubator's ability to support electrical cell testing, such as impedance spectroscopy and electrochemical assays, makes it an attractive option for researchers studying the electrophysiological properties of cells or developing new biosensors and diagnostic devices. Overall, the findings of this study highlight the potential of the mini-incubator as a viable option for cell culture, and further research in this area could yield valuable insights into its advantages, limitations, and applications. To further enhance the mini-incubator system, several suggestions can be considered. One suggestion is the integration of an imaging system directly into the device, creating an integrated platform for cell culture and imaging. This integration would provide researchers with the convenience of real-time monitoring and analysis of cellular processes within the mini-incubator, eliminating the need for separate imaging setups like microscopes. It would streamline the experimental workflow and offer a more compact and efficient solution for cell culture and imaging experiments. Another suggestion is to consider utilizing stainless steel for the interior environment of the mini-incubator. Stainless steel possesses excellent corrosion resistance properties, making it highly suitable for maintaining a sterile and contamination-free environment for cell culture. Its smooth surface facilitates easy cleaning and sterilization procedures, ensuring optimal hygiene.

## Conclusion

In conclusion, the results of this study provide valuable insights into the performance of cell culture in a mini-incubator compared to a standard incubator. The qualitative cell count data demonstrated that the mini-incubator exhibited comparable cell viability and confluency levels to the standard incubator, indicating its suitability for supporting cell growth and proliferation. The flow cytometry analysis revealed a minimal increase in apoptosis levels in the mini-incubator group, suggesting that the mini-incubator may not significantly impact cellular apoptosis. Time-lapse imaging further supported the effectiveness of the mini-incubator in supporting cell growth and division, as visually observed through the movies and images captured at different time points. These findings collectively suggest that the mini-incubator may be a viable alternative for cell culture, providing a favorable environment for cell growth and proliferation.

The results of this study have important implications for various research areas, such as cancer cell culture, drug screening, and other cell-based assays. The mini-incubator could potentially offer advantages in terms of cost-effectiveness, space utilization, and portability, which could benefit researchers with limited resources or space constraints. However, it is important to note that further studies are warranted to fully understand the mechanisms and implications of using the mini-incubator for specific cell types, experimental conditions, and research objectives.

Overall, the findings of this study suggest that the mini-incubator is a promising option for cell culture, and further research in this area could lead to the development of more cost-effective and efficient methods for cell culture, with important implications for biomedical research and applications.

### Supplementary Information

Supplementary Video 1.

Supplementary Video 2.

Supplementary Information 1.

## Data Availability

The data generated and analyzed in this study are available in the published article and its supplementary information files. Supplementary files, Sup1-MDA_MB_231.mp4, Sup2_VERO.mp4, and Sup3_CAD.pdf provide access to the time-lapse imaging results and CAD material.
