# Supplementary material for: Facilitating long-term cell examinations and time-lapse recordings in cell biology research with CO2 mini-incubators
Source: Sci Rep. 2024 Feb 10;14:3418. doi: 10.1038/s41598-024-52866-y (PMC10858865; doi:10.1038/s41598-024-52866-y)
Supplement: Supplementary file 3 — Supplementary Information 1. [file 41598_2024_52866_MOESM3_ESM.pdf]

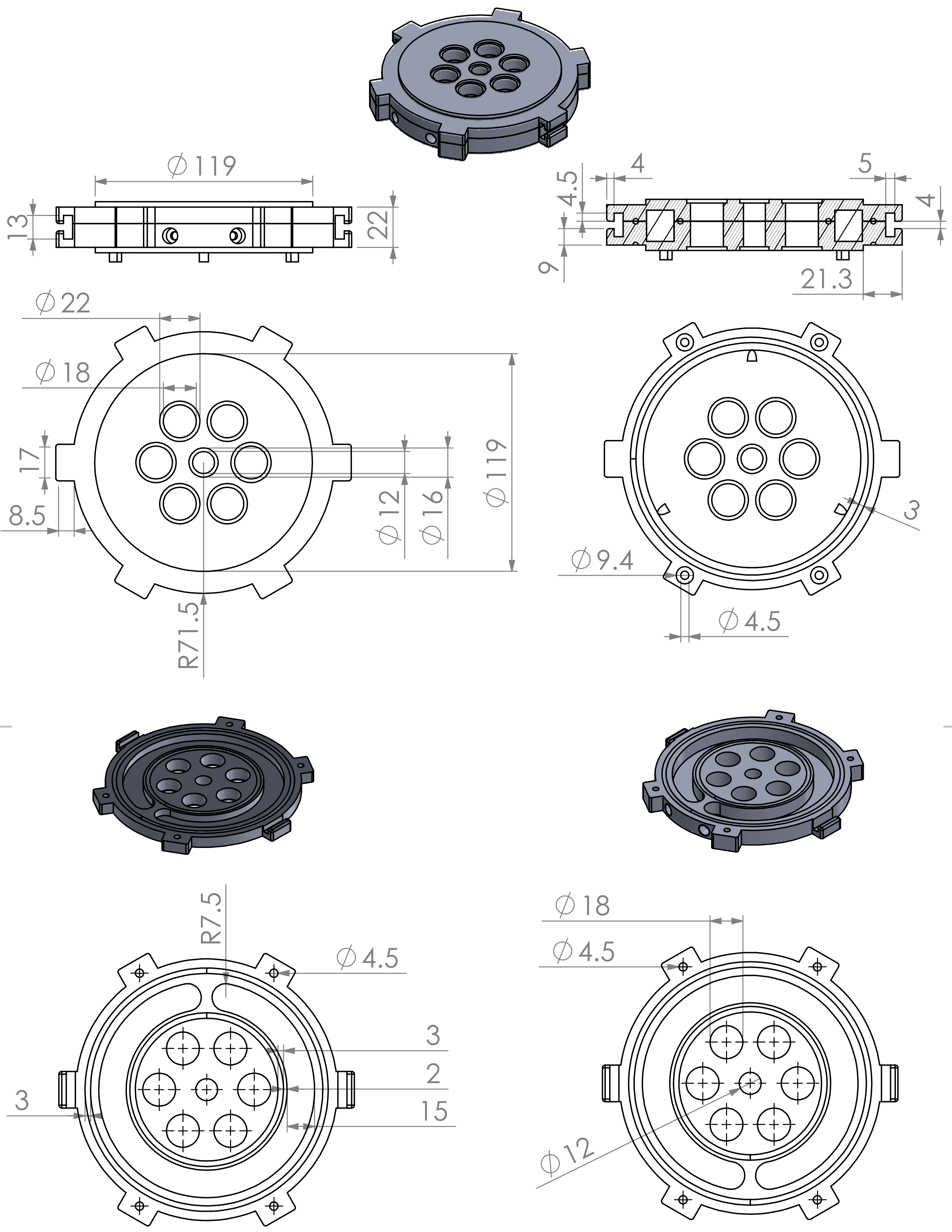

DESIGNED BY: ALI TALEBIPOUR<br>DATE: 11-20-2021		**Mini-Incubator**<br>**MATERIAL: Aluminium Alloy 6061**	
SIZE A4			
SCALE 1:3	WEIGHT	DRAWING NUMBER Part 1 of 2	SHEET 1 of 2

This drawing is our property; It can't be reproduced or communicated without our written consent.

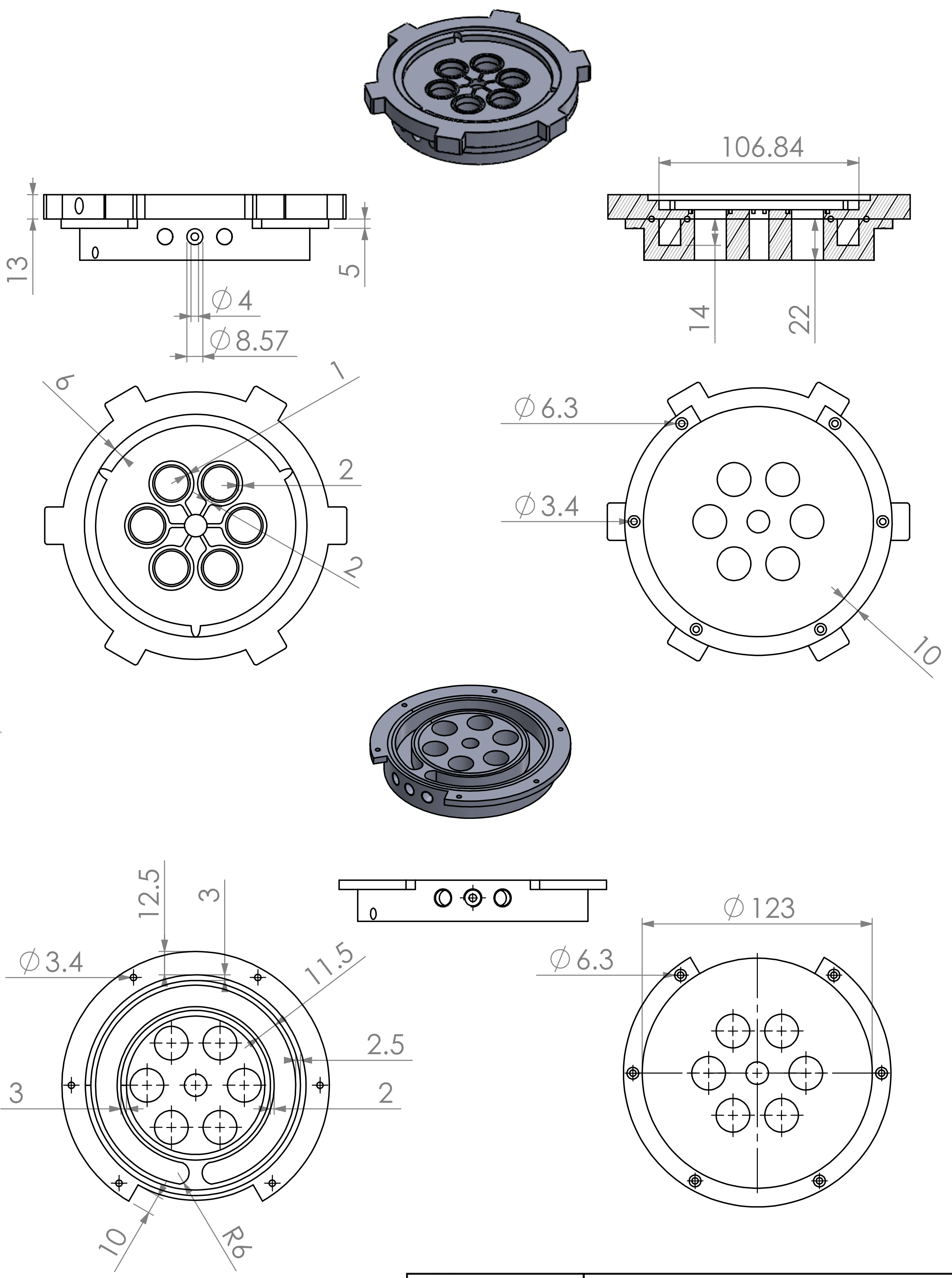

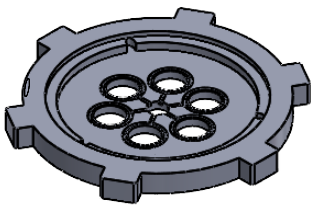

DESIGNED BY: ALI TALEBIPOUR<br>DATE: 11-20-2021	**Mini-Incubator**<br>**MATERIAL:** Aluminium Alloy 6061		
SIZE **A4**			
SCALE<br>1:3	WEIGHT	DRAWING NUMBER<br>Part 2 of 2	SHEET<br>2 of 2

This drawing is our property; It can't be reproduced or communicated without our written consent.
